# PKCδ silencing alleviates saturated fatty acid induced ER stress by enhancing SERCA activity

**DOI:** 10.1042/BSR20170869

**Published:** 2017-11-23

**Authors:** Shujie Lai, Yan Li, Yi Kuang, Hongli Cui, Yang Yang, Wenjing Sun, Kaijun Liu, Dongfeng Chen, Qixian Yan, Liangzhi Wen

**Affiliations:** 1 Department of Gastroenterology, Institute of Surgery Research, Daping Hospital, Third Military Medical University, Chongqing 400042, China; 2 Department of Gastroenterology, The 42 Hospital of People’s Liberation Army, Sichuan 614199, China; 3 Department of Hepatobiliary Surgery, Institute of Surgery Research, Daping Hospital, Third Military Medical University, Chongqing 400042, China

**Keywords:** calcium homeostasis, ERS, NAFLD, PKCδ, SERCA

## Abstract

Protein kinase C δ (PKCδ) plays an important role in nonalcoholic fatty liver disease (NAFLD), however, the mechanism remains unknown. The present study explored the role of PKCδ in NAFLD development and investigated the relationships between PKCδ, calcium homeostasis, and endoplasmic reticulum (ER) stress (ERS). Hepatic steatosis cell model was induced by palmitic acid (PA) in L02 cells. Lipid accretion was evaluated using Oil Red O staining and a triglyceride (TG) detection kit. PKCδ was down-regulated by siRNA. RT-PCR and Western blotting were used to detect the expression of ERS markers. The fluorescence of Ca^2+^ influx was recorded using confocal microscopy. Sarco-ER Ca^2+^-ATPase (SERCA) activity was measured by ultramicro-ATP enzyme test kit. PA treatment induced lipid accretion in L02 cells, destroyed the ER structure, and increased PKCδ activation in a time-dependent manner. Further, PA treatment significantly increased the expression of ERS markers, Ig heavy chain binding protein (Bip), and homologous proteins of CCAAT-enhancer binding proteins (CHOP). PKCδ silencing down-regulated Bip and CHOP expression, indicating a successful alleviation of ERS. The increased calcium storage induced by PA stimulation was significantly decreased in L02 cells treated with PKCδ siRNA compared with the negative control. Moreover, diminished SERCA activity caused by PA was recovered in PKCδ siRNA transfected cells. To the best of our knowledge, this is the first report demonstrating that the inhibition of PKCδ alleviates ERS by enhancing SERCA activity and stabilizing calcium homeostasis.

## Introduction

Nonalcoholic fatty liver disease (NAFLD) is a leading cause of chronic liver diseases with an increasing prevalence worldwide. NAFLD is often associated with metabolic diseases including obesity, dyslipidemia, and diabetes mellitus [1,2]. NAFLD encompasses a wide range of manifestations ranging from benign steatosis to nonalcoholic steatohepatitis (NASH). The early manifestation of NAFLD is fatty degeneration of the liver characterized by excessive accumulation of triglycerides (TGs) in the hepatocytes [3]. NASH is characterized by liver inflammation that can progress to cirrhosis, a major cause of morbidity and mortality [4]. Accumulating evidence suggests that endoplasmic reticulum (ER) stress (ERS) is an important risk factor associated with NASH development [5].

The disruption of ER function results in the accumulation of unfolded proteins in the ER lumen that induces ERS. Persistent ERS activates apoptotic pathways and triggers hepatocyte damage [6]. Calcium plays a critical role in the development of ERS, as calcium reduction in the ER lumen is known to cause ERS during the pathogenic course of NAFLD [7,8]. Further, free fatty acids induce ERS by inhibiting sarco-ER Ca2+-ATPase (SERCA) function, thereby leading to the disruption of calcium homeostasis [9,10]. Binding Ig protein (Bip, also known as GRP78) is a key regulatory protein that stabilizes ER homeostasis during ERS. Homologous protein of CCAAT-enhancer binding protein (CHOP) is a transcriptional factor that regulates ERS-mediated cell apoptosis and serves as a marker for ERS [11]. The increased expression of activating transcription factor-6 (ATF6) and inositol-requiring protein-1 (IRE-1) is closely related to ERS [12].

The increased expression of novel protein kinase C (PKC) isoforms is a major risk factor associated with the development of diseases caused by fatty acid accumulation [13,14]. PKCδ is encoded by the *PRKCD* gene and is primarily expressed in the liver. Bezy et al. [15] previously reported increased PKCδ levels in obese humans and mice. Furthermore, total or liver-specific knockout of the *PRKCD* gene resulted in improved glucose tolerance and insulin sensitivity in mice. Additionally, the TG levels in the liver, plasma, and adipose tissue of epididymis were decreased in *Prkcd^−/−^* mice [16]. Therefore, PKCδ activation can be closely related to diabetes and its relevant disease pathogenesis. PKCδ participates in the fatty degeneration of hepatocytes during the onset and development of NASH, likely by inducing and maintaining ERS. Furthermore, fatty acids could activate PKCδ, leading to the activation of CHOP-induced hepatocyte apoptosis [17]. Therefore, the present study explored the effects of PKCδ on the structure and function of ER during palmitic acid (PA) induced fatty degeneration in a human hepatic cell line (L02 cells). We down-regulated PKCδ in L02 cells and analyzed the relationship between PKCδ and SERCA during PA-induced ERS in L02 cells.

## Methods

### Cell culture and treatment

The human hepatic cell line L02 was obtained from the Cell Bank of the Institute of Biochemistry and Cell Biology (Shanghai, China). L02 cells were cultured in RPMI-1640 medium (GIBCO, Grand Island, NY) supplemented with 10% FBS (GIBCO, Grand Island, NY), 50 U/ml penicillin, and 50 µg/ml streptomycin (Sigma, St. Louis, MO, U.S.A.) and incubated at 37°C in a 5% CO_2_ humidified atmosphere. L02 cells were incubated with 0.5 mM PA (PA group) or equivalent amounts of fatty acid free BSA (control group) for 0, 8, 16, and 24 h.

### siRNA transfection

siRNA was used to specifically target PKCδ. L02 cells were transiently transfected with PKCδ siRNA (Genesis Biotechnology, Wuhan, China) or negative control siRNA (Santa Cruz, CA, U.S.A.) according to the standard protocols. Briefly, cells were seeded at 2 × 10^5^ in 12-well plates without antibiotics. Next, cells with 30–50% confluence were transfected with 50 nM PKCδ siRNA or negative control using Lipofectamine 2000 (Invitrogen, U.S.A.) according to the manufacturer’s protocol. The medium was replaced with fresh culture medium without antibiotics after 4–6 h. Next, the medium was replaced with normal culture medium after 24 h, and subsequent treatments were performed with PA or fatty acid free BSA as described above.

### Oil Red O staining

The Oil Red O stock solution was composed of 5 g Oil Red O in 100 ml isopropyl alcohol. Fresh Oil Red O working solution was prepared by diluting the Oil Red O stock with distilled water in a 3:2 ratio. Cells were washed twice in PBS after PA treatment, fixed with 4% formaldehyde for 30 min, and stained with Oil Red O working solution for 40 min at room temperature. Then, L02 cells were washed with 60% isopropyl alcohol once followed by washing with water twice. The nuclei of the cells were stained with Hematoxylin. The number of Oil Red O-positive cells was counted in replicates in 15 randomly selected fields at 20× magnification under a bright-field microscope.

### Quantitation of TG levels in L02 cells

L02 cells were washed twice with PBS after PA or fatty acid free BSA treatment. Next, L02 cells were lysed on ice with RIPA buffer (Beyotime, Beijing, China) for 30 min and centrifuged at 13000 ***g*** for 20 min at 4°C. The supernatant was then transferred into a new tube. The protein concentration was determined using the BCA method, and the TG levels were determined using a TG kit (Applygen, Beijing) according to the manufacturer’s instructions. The lipid content in L02 cells was defined as micrograms of TG per milligram of the total protein.

### TEM

L02 cells were harvested, fixed with 2.5% glutaraldehyde, washed twice with PBS for 30 min, fixed with 2.5% osmium tetroxide, and stained with 4% aqueous uranyl acetate. The cells were dehydrated with a graded series of acetone, embedded in epoxy resin, and excised into semithin and ultrathin sections. Electron micrographs were performed in the Central Laboratory of Third Military Medical University using a transmission electron microscope (FEI, U.S.A.).

### Assessment of calcium levels in L02 cells

At 70–80% confluence, L02 cells were treated with PA for 24 h. Next, the cells were washed with PBS buffer, incubated with Fluo-4/AM (Abcam, Cambridge, U.K., final concentration: 5 µM) and Pluronic F-127 (Abcam, Cambridge, U.K., final concentration: 0.035%) for 1 h at 37°C and protected from light. Finally, KCl (20 mM) was added to open the voltage-gated calcium channels to induce the consumption of calcium storage in the ER. The fluorescence of Ca^2+^-influx levels in the Fluo-4/AM-treated cells was recorded using confocal microscopy (excitation at 494 nm and emission at 516 nm).

### Real-time PCR

Total RNA was isolated from L02 cells using TRIzol (TaKaRa) following the standard protocol. Primers were designed using Primer 5. The primer sequence and the RT-PCR cycle conditions are summarized in Supplementary Table S1. Real-time qPCR was performed using an Mx3000 PCR machine (Stratagene) and SYBR Premix Ex Taq™ kit (TaKaRa).

### Western blot

Following PA or fatty acid free BSA treatment, L02 cells were washed with PBS and lysed using RIPA lysis buffer (Cell Signaling, Danvers, MA). Lysates were centrifuged for 10 min at 13000 ***g***, and the protein concentration was determined using a BCA assay. Proteins were separated via SDS/PAGE (12% gel) and transferred on to nitrocellulose membrane (HAHY00010, Millipore). Membranes were blocked in PBS-T containing 5% skim milk/BSA for 2 h prior and incubated with the primary antibody overnight at 4°C. The primary antibodies included rabbit polyclonal antibody against PKCδ, p-PKCδ, Bip, CHOP (1:1000; Cell Signaling Technology, U.S.A.), β-actin (1:1000; Biosynthesis Biotechnology, Beijing, China), ATF6 and goat polyclonal antibody IRE-I (1:1000; Origene Technology, U.S.A.). After 2-h incubation with the corresponding secondary antibody (1:10000; donkey-anti-mouse or donkey-anti-rabbit, IRDye 700 or IRDye 800, respectively), signals were detected using an Odyssey infrared imaging system at a wavelength of 700 or 800 nm.

### SERCA activity analysis

To transport calcium from the cytosol into the ER lumen, SERCA catalyzes the hydrolysis of ATP to ADP and inorganic phosphate. Therefore, the amount of inorganic phosphate reflects SERCA activity. SERCA activity was analyzed using the ultramicro ATP enzyme test kit as described previously [18]. Following trypsinization and centrifugation, L02 cells were resuspended in normal saline and lysed by sonication. Protein concentration was determined by the BCA method. The activity of the supermicro Ca^2+^-ATP enzyme was measured using a commercially available kit (Biological Technology, Beijing, China) according to the manufacturer’s instructions. The specific activity of SERCA was expressed as µmol Pi/mg prot/h.

### Statistical analysis

The differences between groups were analyzed using Student’s *t* test. All the data are presented as the mean ± S.D. Data were analyzed using the statistics software SPSS 18.0. A *P*-value <0.05 was considered to be statistically significant.

## Results

### PA increased the accumulation of lipid droplets and TG in L02 cells

**Figure 1:**
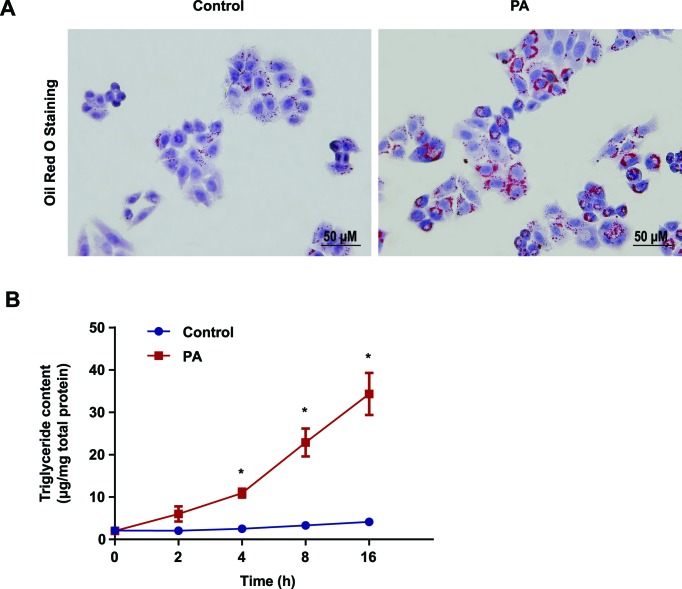
PA increased lipid droplets and TG accumulation in L02 cells (**A**) Oil Red O staining was performed in L02 cells after incubating with BSA (control) or 0.5 mM sodium palmitate (PA) for 16 h. (**B**) L02 cells were treated with 0.5 mM PA for 2, 4, 8, and 16 h to measure TG levels with an enzymatic assay kit. All the data were repeated three times. Data represent the mean ± S.D. *P<0.05.

PA is a saturated fatty acid that can induce fatty degeneration in hepatocytes [19,20]. To evaluate the effect of PA on fatty degeneration in L02 cells, we pretreated the L02 cells with PA (PA group) or BSA (control group) for 0, 2, 4, 8, and 16 h. Oil Red O staining demonstrated a higher accumulation of lipid droplets in PA-treated cells compared with the control group (Figure 1A and Supplementary Figure S1). In the PA-treated group, the TG content increased in a time-dependent manner (Figure 1B). Following PA treatment, we observed a significant difference in TG content between the control and PA-treated group at 0 h (2.11 ± 0.20 compared with 2.04 ± 0.59 µg/mg), 2 h (2.06 ± 0.24 compared with 6.02 ± 1.79 µg/mg), 4 h (2.53 ± 0.37 compared with 10.94 ± 1.04 µg/mg), 8 h (3.33 ± 0.38 compared with 22.90 ± 3.28 µg/mg ), and 16 h (4.14 ± 0.42 compared with 34.36 ± 4.95 µg/mg) (*P*<0.05; Figure 1B).

### PA destroyed the structure and integrity of ER

**Figure 2:**
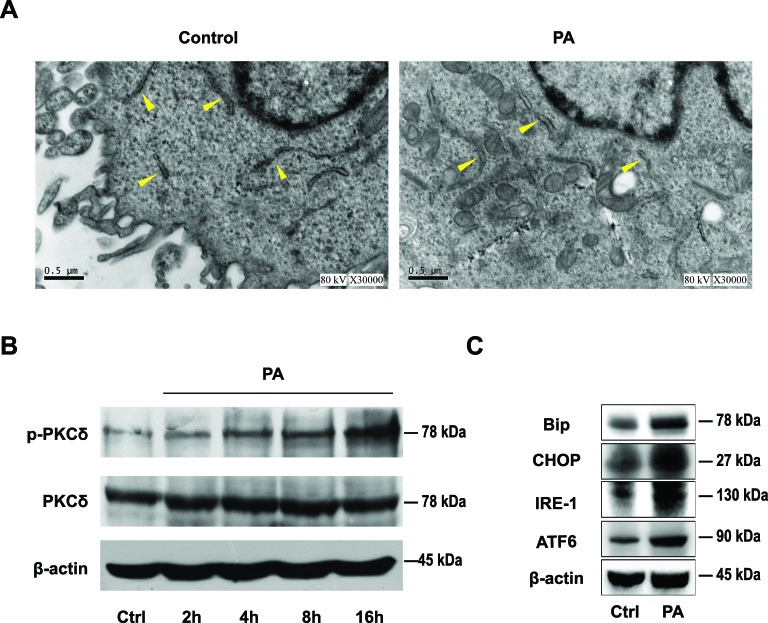
PA destroyed the integrity of ER and up-regulated the expression of Bip, CHOP, and p-PKCδ (**A**) L02 cells were incubated with BSA or 0.5 mM PA for 16 h, and the morphological changes were observed by TEM. Arrowheads indicate the rough ER. (**B**) Western blotting analysis showed PKCδ and p-PKCδ expression at the indicated time with or without PA treatment. (**C**) Western blotting analysis indicated Bip, ATF6, IRE-1, and CHOP expression in L02 cell treated with BSA or PA for 16 h. All the data repeated three times.

Following a 16-h PA or BSA treatment, we determined the ultrastructure of the ER using EM. In BSA-treated control group, the rough ER maintained its normal structure with ribosomes adhering to the surface (Figure 2A, left panel). In contrast, PA treatment destroyed the structure and integrity of ER as evidenced by rough ER dilatation and degranulation (Figure 2A, right panel).

### PA increased p-PKCδ and induced ERS

Following PA treatment, we determined PKCδ activation by Western blotting in L02 cells. The p-PKCδ (Thr^505^) was up-regulated in the PA-treated group compared with the control group in a time-dependent manner, while the expression of PKCδ remained constant (Figure 2B). Further, we investigated the expression of ERS markers to determine the effect of PA on triggering ERS in L02 cells. The up-regulation of Bip, CHOP, ATF6, and IRE-1 were observed in the PA-treated group compared with the control group (Figure 2C). These results indicate the activation of ERS in L02 cells by PA treatment. Further, lipid accretion induced the activation of PKCδ, which may play a crucial role in NAFLD development.

### Inhibition of PKCδ alleviates PA-induced ERS

**Figure 3:**
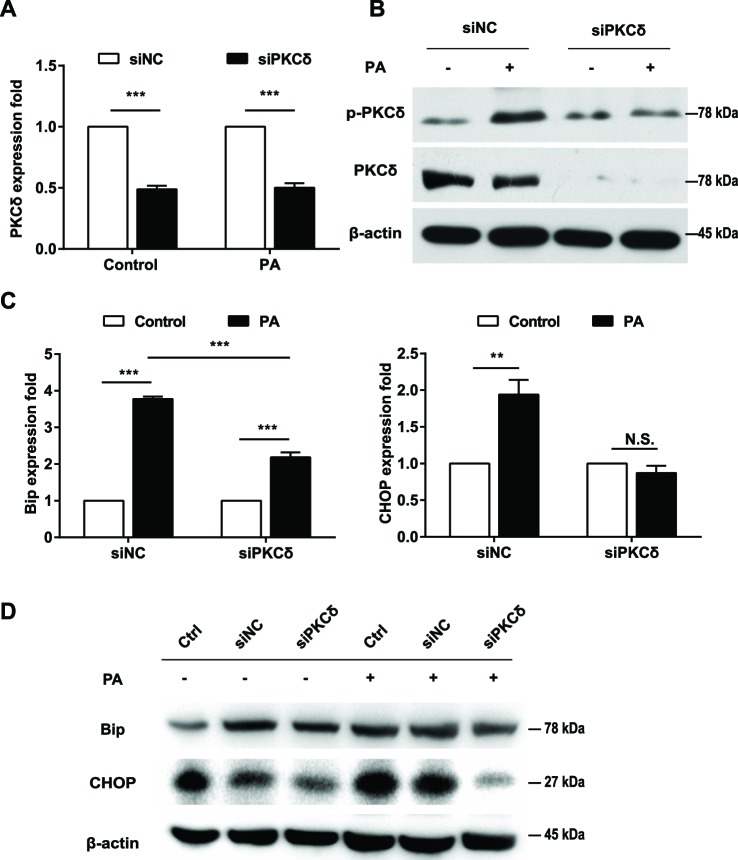
PKCδ silencing protected L02 cells from PA-induced ERS L02 cells were transfected with PKCδ siRNA (siPKCδ) or negative control siRNA (siNC) for 24 h and then treated with BSA or 0.5 mM PA for an additional 16 h. Real-time PCR showed the expression of *PKCδ* (**A**), *Bip* and *CHOP* (**C**). (**B**) Western blots showed the expression of PKCδ and p-PKCδ. (**D**) Western blots showed the expression of Bip and CHOP in L02 cells with siPKCδ or other indicated treatments. All the data repeated three times. Data represent the mean ± S.D. **P<0.01, ***P<0.001. Abbreviation: N.S., no significance.

Next, we investigated the impact of PKCδ inhibition on PA-induced ERS in L02 cells. Compared with the control siRNA, the expression of PKCδ was significantly decreased in L02 cells following PKCδ siRNA treatment (*P*<0.001; Figure 3A,B). Additionally, the p-PKCδ was inhibited in the siPKCδ-treated group compared with the negative control group in PA-treated L02 cells (Figure 3B). Our results showed that PKCδ silencing inhibited lipid accumulation of L02 cells induced by PA (Figure S3A, B). In the control siRNA group, PA treatment resulted in the up-regulation of Bip and CHOP expression (*P*<0.01, Figure 3C,D). However, the down-regulation of PKCδ by siRNA significantly decreased the effect of PA on Bip and CHOP (Figure 3C,D, FigureS3C, D). Notably, the PA-induced up-regulation of CHOP was completely abolished in the PKCδ siRNA-treated group (Figure 3D). These results indicate that the ERS in PA-treated L02 cells was impeded by PKCδ silencing.

### PKCδ silencing maintains calcium homeostasis in the ER

**Figure 4:**
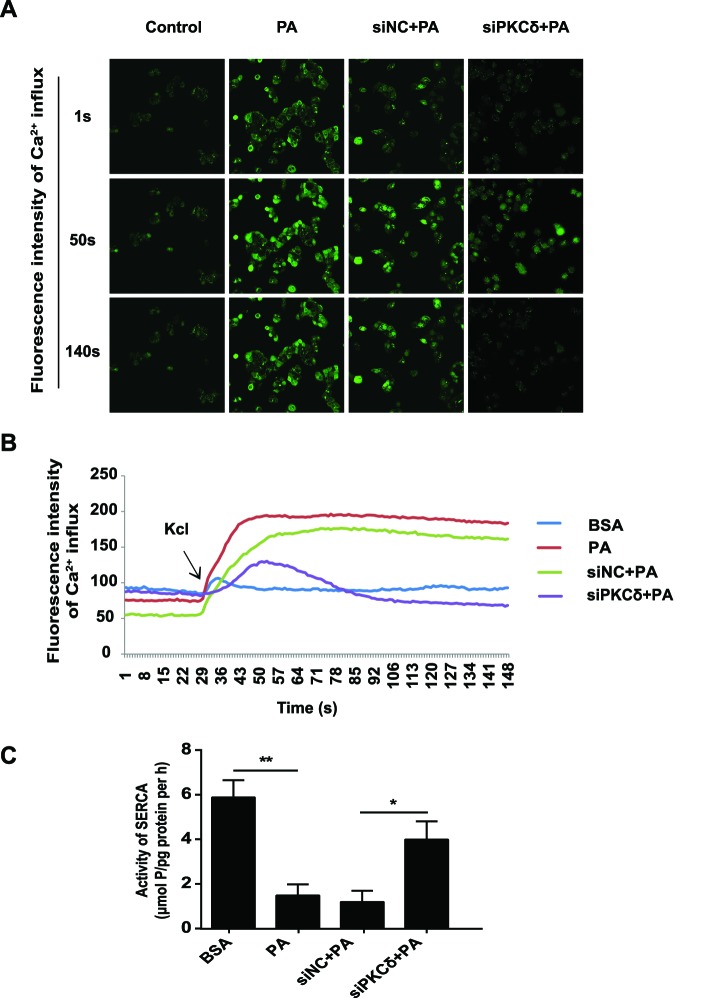
PKCδ silencing maintained the balance of Ca^2+^ homeostasis and increased the activity of SERCA (**A, B**) L02 cells were incubated with BSA or PA for 16 h; 20 mM KCl was then added to activate store-operated Ca^2+^ entry. The fluorescence densities of Ca^2+^ change were monitored in Fluo-4/AM-loaded L02 cells. (**C**) Measurement of the SERCA activity in L02 cells with different pretreatments at 16 h after PA treatment. All the data repeated three times. Data represent the mean ± S.D. *P<0.05, **P<0.01.

Accumulating evidence demonstrated that calcium imbalance in ER is crucial for the development of ERS. In the present study, we used Fluo-4/AM, a calconcarboxylic acid dye, to determine changes in the calcium concentrations in L02 cells during ERS. We used a high concentration of KCl to open the voltage-gated calcium channels to induce the consumption of calcium storage in ER [21]. PA treatment increased calcium storage. This increase was partially attenuated upon the down-regulation of PKCδ compared with the negative control (Figure 4A,B). This indicates that PKCδ down-regulation during ERS can cause the desensitization of L02 cells to PA-induced calcium imbalance to protect against the exhaustion of calcium stores in the ER.

### PKCδ silence recovers SERCA activity

SERCA is a pivotal factor in the maintenance of ER calcium balance. Therefore, we explored the potential protective effect of PKCδ silencing on SERCA activity. Compared with the BSA treatment, SERCA activity was inhibited in L02 cells treated with PA for 16 h. However, the down-regulation of PKCδ successfully restored the SERCA activity caused by PA (Figure 4C, Figure S4).

## Discussion

The excessive accumulation of lipids in the liver leads to the development of NAFLD, which can progress to NASH. However, the exact mechanism underlying NASH development has not been fully elucidated. ERS plays a pivotal role in NASH development, aggravation of insulin resistance, and inflammatory responses by inducing hepatocyte apoptosis [22–24]. The disruption of calcium homeostasis and the accumulation of misfolded proteins are known risk factors associated with ERS. Long-term ERS often causes apoptosis, and promotes lipid synthesis and inflammatory reactions. Previous studies demonstrated that PA can induce lipid accumulation and possibly trigger ERS by changing calcium flux from ER to cytoplasm before inducing apoptosis [25]. Therefore, the present study used PA to induce NAFLD and fatty degeneration in the human hepatocyte L02 cells and investigated the underlying molecular mechanisms. In agreement with previous reports [26], Oil Red O staining confirmed the increase of lipid droplets in L02 cells cultured in PA (0.5 mM) and elevated the TG content. TEM verified the swelling of the ER lumen and the denudation of ribosomes after 16-h stimulation with PA. These results indicated that PA treatment successfully induced fatty degeneration in L02 hepatocytes. And in the present study, we found that Bip, ATF6, IRE-1, and CHOP expression were increased in the PA-treated group, indicating PA triggered ERS in L02 cells.

PKCδ has a recently recognized role in the pathological manifestations of fatty liver disease [15]. The PKCδ subtype has a high affinity toward diacylglycerol, a metabolite for free fatty acids [27]. Itani et al. [28] demonstrated that lipid perfusion can induce the activation of PKCδ and PKCθ isoforms in the muscle and liver, rather than the classical PKC subtypes. Further, Samuel et al. [29] proved that the reduction in PKC expression in the liver and white adipose tissue prevented fat-induced fatty degeneration of hepatocytes. In addition, Frangioudakis et al. [16] showed that PKCδ-deficient mice could successfully reduce the hyperlipidemia induced by a high-fat diet and had decreased lipogenic enzymes. Notably, in the present study, we observed the activation of PKCδ in L02 cells treated with PA. The phosphorylation of PKCδ at the site of Thr^505^ was up-regulated in a time-dependent manner. In contrast, the down-regulation of PKCδ resulted in the decrease of the p-PKCδ and the down-regulation of Bip and CHOP. Therefore, it can be speculated that PKCδ plays an important role in ERS.

ER maintains intracellular calcium homeostasis. Under normal conditions, ER secretes Ca^2+^ from the ER lumen to the cytoplasm primarily by ryanodine receptor and inositol 1,4,5-trisphosphate receptor mediated pathways and uptakes Ca^2+^ from the cytoplasm into the ER lumen by SERCA to maintain a dynamic balance of Ca^2+^ in the ER. The reduction in SERCA activity increases the cytosolic calcium and promotes apoptosis [30–32]. In *ob/ob* mice, the disruption of SERCA resulted in ERS and insulin resistance, indicating the important role of SERCA in metabolic disorders [33]. SERCA2 deficiency occurs in human Darier–White disease, leading to glucose intolerance, decreased insulin secretion, reduced β-cell proliferation, and increased β-cell ERS [34]. SERCA2 deficiency leads to activation of β-cell ERS [35]. Broad-spectrum PKC inhibitors were found to increase the Ca^2+^ concentration induced by thrombin in platelets. However, our knowledge of the mechanism and physiological significance of these signaling pathways remains limited [36].

The present study observed that intracellular calcium homeostasis in L02 cells was significantly altered after PA treatment, whereas intracellular calcium homeostasis was nearly maintained in siPKCδ-transfected cells following PA exposure. Therefore, PKCδ silencing in L02 cells may have played a pivotal role in maintaining calcium homeostasis. Previous research demonstrated the reduction in SERCA expression and high ERS levels in hepatocytes and macrophages of obese mice along with insulin resistance [37]. Upon the recovery of normal SERCA function, ERS was relieved, glucose homeostasis and insulin sensitivity were improved, and the hepatic fatty production and TG accumulation were decreased. Similarly, in the present study, PA triggered ERS by inhibiting SERCA activity and induced a severe overload of calcium that resulted in the fatty degeneration of L02 cells. In contrast, PKCδ silencing or inhibition relieved the calcium overload in L02 cells by increasing SERCA activity. Therefore, the down-regulation of PKCδ can provide effective protection for ERS-induced hepatocyte death.

In summary, PKCδ silencing could down-regulate Bip and CHOP and alleviate ERS by recovering SERCA activity. These results indicated that PKCδ played an important role in the development of NAFLD. However, the exact mechanism by which PKCδ regulates SERCA activity remains largely unknown. Nevertheless, to the best of our knowledge, this is the first report demonstrating that the inhibition of PKCδ alleviates ERS by enhancing SERCA activity and stabilizing calcium homeostasis.

## Supporting information

## Abbreviations

ATF6: activating transcription factor-6
Bip: binding Ig protein
CHOP: homolugous protein of CCAAT-enhancer binding protein
ER: endoplasmic reticulum
ERS: ER stress
IRE-1: inositol-requiring protein-1
NAFLD: nonalcoholic fatty liver disease
NASH: nonalcoholic steatohepatitis
PA: palmitic acid
PKCδ: protein kinase C δ
RT-PCR: real-time PCR
SERCA: sarco-ER Ca^2+^-ATPase
TG: triglyceride
