# Supplementary figures and images for: PKCδ silencing alleviates saturated fatty acid induced ER stress by enhancing SERCA activity
Source: Biosci Rep. 2017 Nov 23;37(6):BSR20170869. doi: 10.1042/BSR20170869 (PMC5700272; doi:10.1042/BSR20170869)

**Oil Red O Staining area (%)**

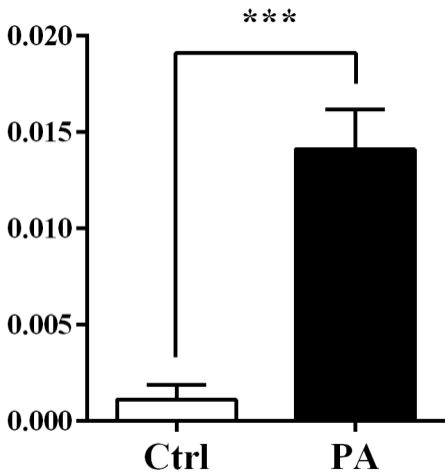

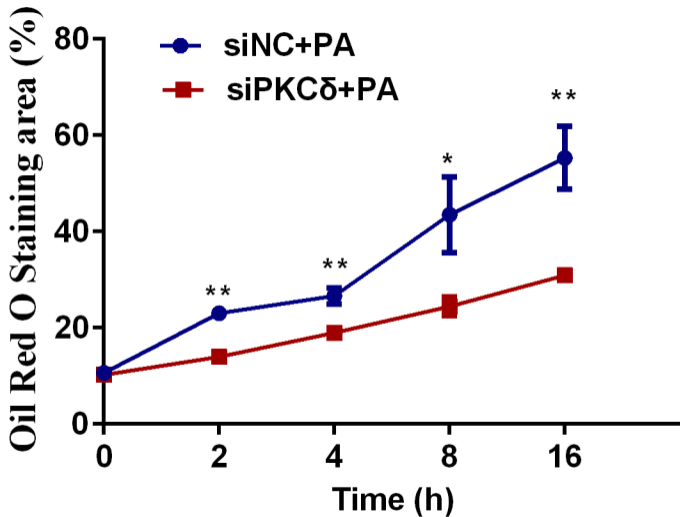

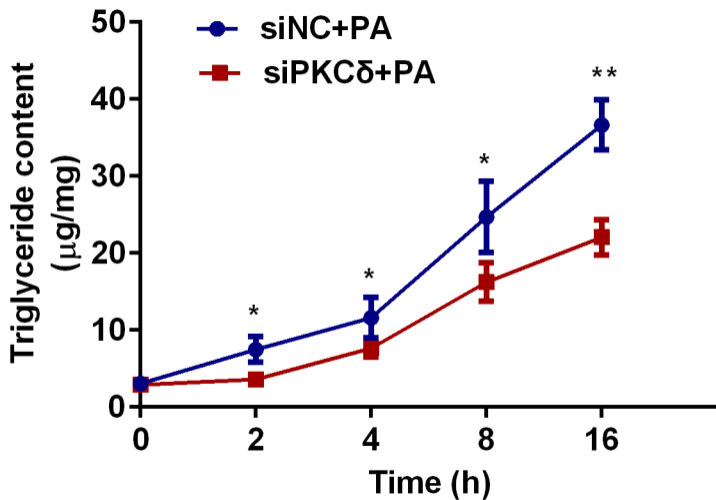

Ctrl siNC siPKC $\delta$

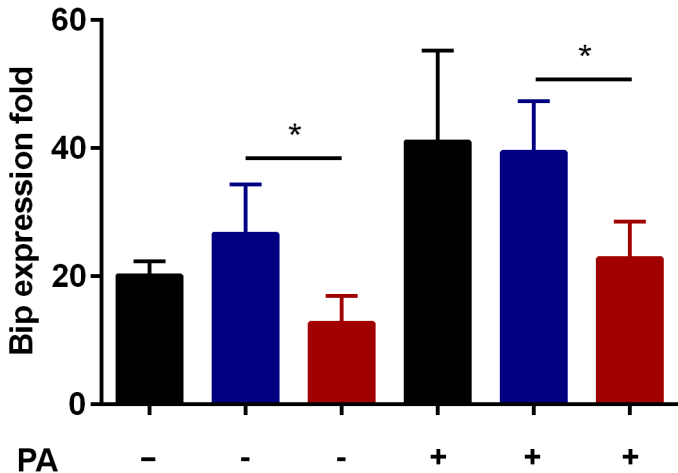

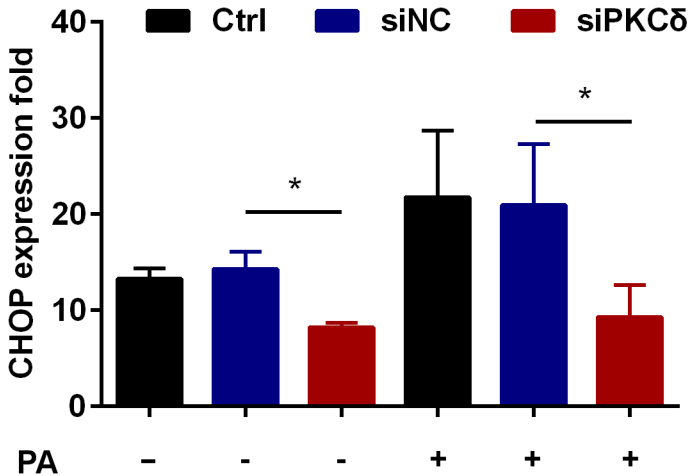

BSA PA siNC+PA siPKC $\delta$ +PA

SERCA2

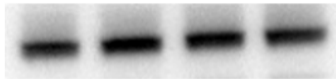

—114 kDa

B-actin

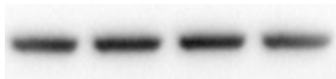

—45 kDa

Supplement: Supplementary file 1 [file bsr20170869_Supp1.pdf]
